# Crystal structure and Hirshfeld surface analysis of 6-((*E*)-2-{4-[2-(4-chloro­phen­yl)-2-oxoeth­oxy]phen­yl}ethen­yl)-4,5-di­hydro­pyridazin-3(2*H*)-one

**DOI:** 10.1107/S205698902101238X

**Published:** 2022-01-01

**Authors:** Said Daoui, Israa Muwafaq, Emine Berrin Çınar, Abdulmalik Abudunia, Necmi Dege, Noureddine Benchat, Khalid Karrouchi

**Affiliations:** a Laboratory of Applied Chemistry and Environment (LCAE), Faculty of Sciences, Mohammed I University, 60000 Oujda, Morocco; b Department of Physics, Faculty of Arts and Sciences, Ondokuz Mayıs University, Samsun, 55200, Turkey; c Department of Pharmacology, Faculty of Clinical Pharmacy, University of Medical and Applied Sciences, Yemen; d Laboratory of Analytical Chemistry and Bromatology, Faculty of Medicine and Pharmacy, Mohammed V University in Rabat, Morocco

**Keywords:** crystal structure, di­hydro­pyridazin, Hirshfeld surface, hydrogen bonding

## Abstract

The pyridazine ring in the title compound, C_20_H_17_ClN_2_O_3_, adopts a screw-boat conformation. The whole mol­ecule is flattened, the dihedral angles subtended by the least-squares plane of the central aromatic ring with those of the terminal benzene and pyridazine rings being 15.18 (19) and 11.23 (19)°, respectively. In the crystal, the mol­ecules are linked by pairs of N—H⋯O bonds into centrosymmetric dimers and by C—H⋯π contacts into columns. The results of the Hirshfeld surface analysis show that the most prominent inter­actions are H⋯H, accounting for 36.5% of overall crystal packing, and H⋯O/O⋯H (18.6% contribution) contacts.

## Abstract 2

The pyridazine ring in the mol­ecule of the title compound adopts a screw-boat conformation. The whole mol­ecule is flattened, the dihedral angles subtended by the least-suares plane of the central aromatic ring with those of the terminal benzene and pyridazine rings being 15.18 (19) and 11.23 (19)°, respectively. In the crystal, the mol­ecules are linked by pairs of N—H⋯O bonds into centrosymmetric dimers and by C—H⋯π contacts into columns.

## Chemical context

Pyridazinone derivatives are a class of nitro­genous heterocyclic compounds that have attracted considerable attention because of their prospective pharmacological and medicinal properties as anti-inflammatory (Boukharsa *et al.*, 2018 ▸), anti­tumor (Bouchmaa *et al.*, 2018 ▸, 2019 ▸), anti­fungal (Rozada *et al.*, 2020 ▸), anti­depressant (Boukharsa *et al.*, 2016 ▸), anti­tubercular, anti­convulsant (Asif *et al.*, 2020 ▸) and anti­viral (El-Shanbaky *et al.*, 2021 ▸) agents. In addition, pyridazinones demonstrate some inter­esting physicochemical properties (Daoui *et al.*, 2020*a* ▸; El Kalai *et al.*, 2021*a* ▸,*b* ▸) and some studies have shown that these compounds are good corrosion inhibitors (Chelfi *et al.*, 2020 ▸). Encouraged by the bioactivity of these compounds and in a continuation of our studies in the field of the synthesis, mol­ecular structures and Hirshfeld surfaces analyses of new pyridazin-3(2*H*)-one derivatives (Daoui *et al.*, 2020*b* ▸, 2021 ▸), we report herein the crystal structure and the results of the Hirshfeld surface analysis of 6-((*E*)-2-{4-[2-(4-chloro­phen­yl)-2-oxoeth­oxy]phen­yl}ethen­yl)-4,5-di­hydro­pyridazin-3(2*H*)-one. 
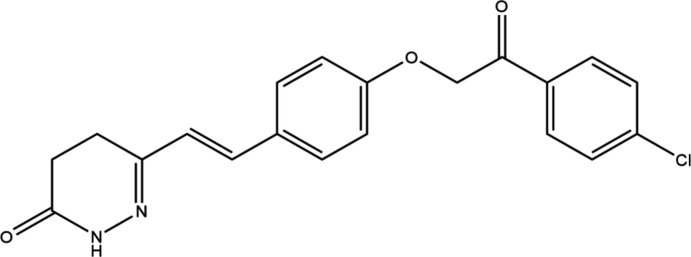


## Structural commentary

**Figure 1:**
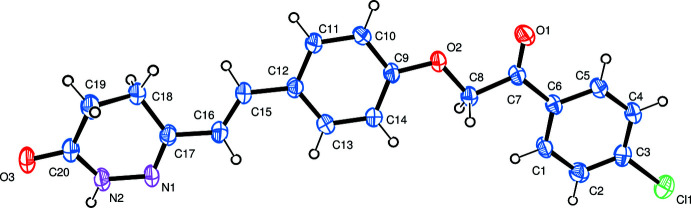
Mol­ecular structure of the title compound showing the atom labelling and displacement ellipsoids drawn at the 50% probability level.

The mol­ecular structure of the title compound is presented in Fig. 1 ▸. The bond lengths in the N1—C15 chain (Table 1 ▸) are consistent with an alternation of double and single bonds while those in the amide fragment indicate strong π-conjugation. The N1—N2 distance of 1.406 (4) Å agrees well with the values for related pyridazinones (Daoui, Çınar *et al.*, 2019 ▸; Daoui, Baydere *et al.*, 2019 ▸). The conformation of the di­hydro­pyridazine ring is close to a screw-boat [Θ = 111.9 (6)°, φ = 34.6 (6)°]. The whole mol­ecule is flattened with the largest deviations from the least-squares plane of 0.356 (4) and 0.339 (5) Å being observed for atoms C18 and C19, respect­ively. The central benzene ring forms dihedral angles of 11.23 (19) and 15.18 (19)° with the planes of the terminal di­hydro­pyridazine and benzene rings, respectively.

## Supra­molecular features

**Figure 2:**
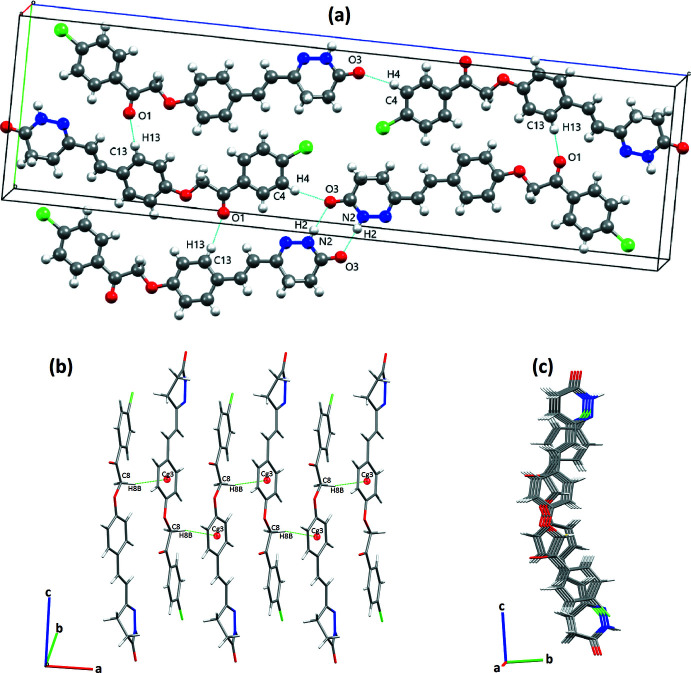
(*a*) A view of the crystal packing of the title compound along the *c* axis. Dashed lines indicate hydrogen bonds. (*b*) C—H⋯π inter­actions. (*c*) A view of the mol­ecular stacks running along the *a* axis.

In the crystal, the mol­ecules are linked into centrosymmetric dimers by pairs of N—H⋯O hydrogen bonds, giving rise to an 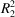 (8) graph-set motif (Fig. 2 ▸ *a*, Table 2 ▸). No π–π inter­actions are present in this structure, but the mol­ecules are connected by weak C—H⋯π contacts into stacks running along the *a-*axis direction (Fig. 2 ▸ *b*,*c*, Table 2 ▸). Other contacts of the C—H⋯O and C—H⋯Cl types further stabilize the crystal structure (Table 2 ▸).

## Hirshfeld surface analysis

**Figure 3:**
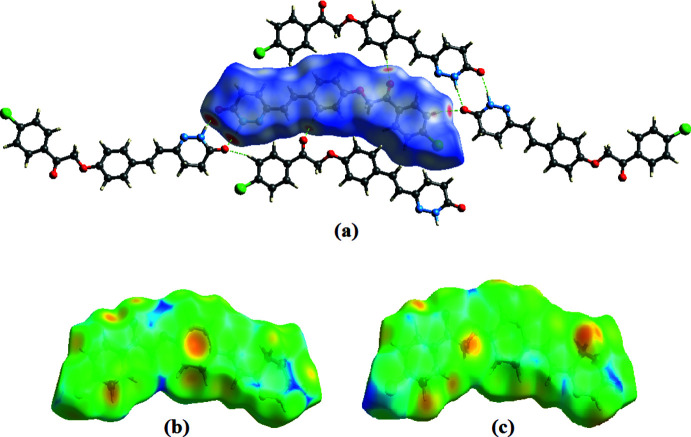
(*a*) Hirshfeld surfaces of the title mol­ecule mapped over (*a*) *d*_norm_, (*b*) *d*_e_ and (*c*) *d*_i_.

In order to visualize and study the inter­molecular contacts, a Hirshfeld surface analysis of the title compound was undertaken using *Crystal Explorer 17.5* (Turner *et al.*, 2017 ▸). Fig. 3 ▸ *a* shows the 3D surface mapped over *d*_norm_ over the range −0.484 (red) to 1.403 (blue) a.u. The pale-red spots on the surface represent short N—H⋯O and C—H⋯O inter­actions (Table 2 ▸). The surfaces mapped over *d*_e_ and *d*_i_ are presented in Fig. 3 ▸ *b* and 3*c*.

**Figure 4:**
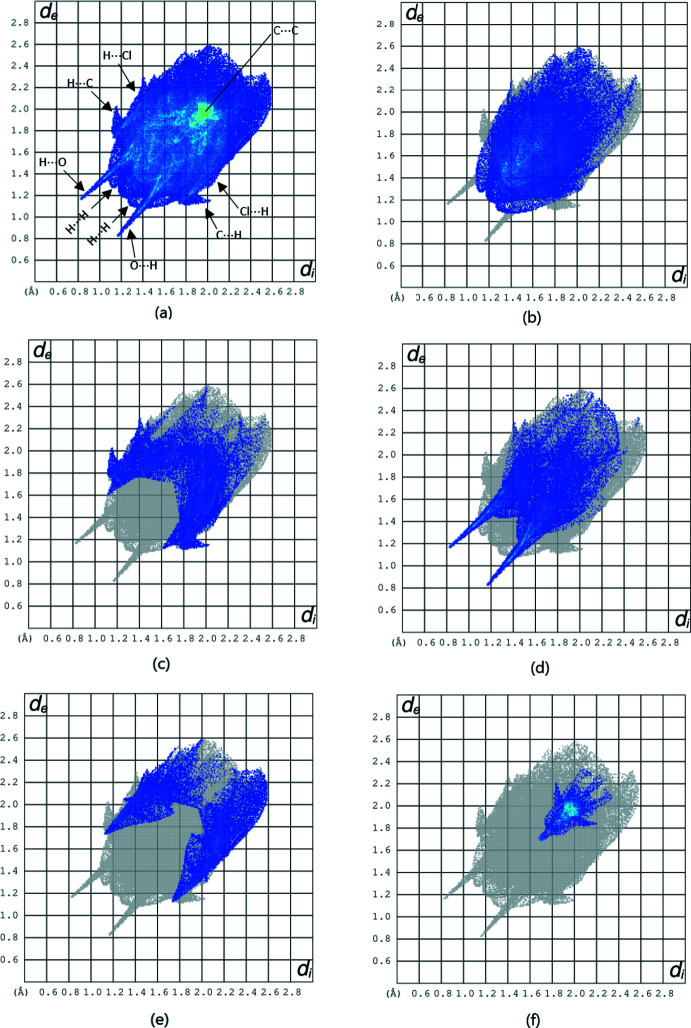
(*a*) The overall two-dimensional fingerprint plot, and those delineated into (*b*) H⋯H, (*c*) H⋯C/C⋯H, (*d*) H⋯O/O⋯H, (*e*) H⋯Cl/Cl⋯H and (*f*) C⋯C inter­actions.

The overall two-dimensional fingerprint plot and those delineated into H⋯H, H⋯C/C⋯H, H⋯O/O⋯H, H⋯Cl/Cl⋯H and C⋯C contacts are presented in Fig. 4 ▸. H⋯H inter­actions are the most prominent, accounting for 36.5% of the overall crystal packing. H⋯O/O⋯H contacts, including inter­molecular C—H⋯O and N—H⋯O hydrogen bonding, make a 18.6% contribution to the Hirshfeld surface. H⋯C/C⋯H contacts add a 15.4% contribution. The contributions from H⋯Cl/Cl⋯H and C⋯C contacts are 11.2% and 7.6%, respectively.

## Database survey

A search of the Cambridge Structural Database (CSD, version 5.40, update March 2020; Groom *et al.*, 2016 ▸) revealed two structures containing the same pyridazinone fragments as in the title structure but with different substituents, *viz*. 6-[(*E*)-2-(thio­phen-2-yl)ethen­yl]-4,5-di­hydro­pyridazin-3(2*H*)-one (MUCLEE; Daoui, Çınar *et al.*, 2019 ▸) and (*E*)-6-(4-hy­droxy-3-meth­oxy­phen­yl)ethenyl-4,5-di­hydro­pyridazin-3(2*H*)-one (LOSSOE; Daoui, Baydere *et al.*, 2019 ▸). Both these structures exhibit bond lengths in the pyridazine ring and N—H⋯O hydrogen-bonding parameters that are very similar to those observed in the title structure.

## Synthesis and crystallization

A mixture of (*E*)-6-(4-hy­droxy­styr­yl)-4,5-di­hydro­pyridazin-3(2*H*)-one (0.5 g, 2.3 mmol), K_2_CO_3_ (0.79 g, 5.7 mmol) and 2-chloro-1-(4-chloro­phen­yl)ethan-1-one (0.47 g, 2.5 mmol) in acetone (50 ml) was refluxed overnight. After cooling, the solution was filtered and the solvent removed under reduced pressure. The residue was purified by recrystallization from ethanol to afford single crystals (yield 72%).

## Refinement

Crystal data, data collection and structure refinement details are summarized in Table 3 ▸. Hydrogen atoms were positioned geometrically and treated as riding, with C—H = 0.96 Å for methyl­ene [*U*_iso_(H) = 1.5 *U*_eq_(C)], C—H = 0.93 Å for aromatic [*U*_iso_(H) = 1.2 *U*_eq_(C)] and C—H = 0.98 Å for methine [*U*_iso_ (H) = 1.2 *U*_eq_(C)] H atoms.

## Supplementary Material

Crystal structure: contains datablock(s) I. DOI: 10.1107/S205698902101238X/yk2160sup1.cif

Structure factors: contains datablock(s) I. DOI: 10.1107/S205698902101238X/yk2160Isup2.hkl

Supporting information file. DOI: 10.1107/S205698902101238X/yk2160Isup3.cml

CCDC reference: 2123627

Additional supporting information:  crystallographic information; 3D view; checkCIF report

## Figures and Tables

**Table 1:** Selected bond lengths (Å)

C20—O3	1.241 (4)	C16—C17	1.459 (4)
N2—C20	1.333 (5)	C15—C16	1.329 (5)
N1—N2	1.406 (4)	C12—C15	1.470 (4)
N1—C17	1.292 (4)	C7—O1	1.219 (4)

**Table 2:** Hydrogen-bond geometry (Å, °) *Cg*3 is the centroid of the C9–C14 ring.

*D*—H⋯*A*	*D*—H	H⋯*A*	*D*⋯*A*	*D*—H⋯*A*
N2—H2⋯O3^i^	0.86	2.11	2.891 (4)	151
C4—H4⋯O3^ii^	0.93	2.44	3.327 (4)	160
C13—H13⋯O1^iii^	0.93	2.53	3.421 (4)	161
C18—H18*A*⋯Cl1^iv^	0.97	2.94	3.737 (3)	140
C8—H8*B*⋯*Cg*3^v^	0.97	2.73	3.514 (3)	138

Symmetry codes: (i) -x+1, -y+2, -z+1; (ii) x, -y+{\script{3\over 2}}, z+{\script{1\over 2}}; (iii) -x+1, y+{\script{1\over 2}}, -z+{\script{3\over 2}}; (iv) -x+1, y-{\script{1\over 2}}, -z+{\script{3\over 2}}; (v) x-{\script{1\over 2}}, y, -z+{\script{3\over 2}}.

**Table 3:** Experimental details

Crystal data	
Chemical formula	C_20_H_17_ClN_2_O_3_
*M*_r_	368.80
Crystal system, space group	Orthorhombic, *P* *b* *c* *a*
Temperature (K)	296
*a*, *b*, *c* (Å)	7.3514 (4), 11.5539 (7), 41.397 (3)
*V* (Å^3^)	3516.2 (4)
*Z*	8
Radiation type	Mo *K*α
μ (mm^−1^)	0.24
Crystal size (mm)	0.45 × 0.20 × 0.05

Data collection	
Diffractometer	Stoe IPDS 2
Absorption correction	Integration (*X-RED32*; Stoe & Cie, 2002 ▸)
*T*_min_, *T*_max_	0.925, 0.994
No. of measured, independent and observed [*I* > 2σ(*I*)] reflections	19519, 2913, 1682
*R*_int_	0.113
(sin θ/λ)_max_ (Å^−1^)	0.584

Refinement	
*R*[*F* ^2^ > 2σ(*F* ^2^)], *wR*(*F* ^2^), *S*	0.060, 0.128, 0.99
No. of reflections	2913
No. of parameters	235
H-atom treatment	H-atom parameters constrained
Δρ_max_, Δρ_min_ (e Å^−3^)	0.34, −0.22

Computer programs: *X-AREA* and *X-RED32* (Stoe & Cie, 2002 ▸), *SHELXT2018/3* (Sheldrick, 2015*a* ▸), *SHELXL2018/3* (Sheldrick, 2015*b* ▸), *OLEX2* (Dolomanov *et al.*, 2009 ▸), *Mercury* (Macrae *et al.*, 2020 ▸), *WinGX* (Farrugia, 2012 ▸), *PLATON* (Spek, 2020 ▸) and *publCIF* (Westrip, 2010 ▸).

## supplementary crystallographic information

### Crystal data

**Table:**

C_20_H_17_ClN_2_O_3_	*D*_x_ = 1.393 Mg m^−^^3^
*M**_r_* = 368.80	Mo *K*α radiation, λ = 0.71073 Å
Orthorhombic, *P**b**c**a*	Cell parameters from 13252 reflections
*a* = 7.3514 (4) Å	θ = 1.0–25.1°
*b* = 11.5539 (7) Å	µ = 0.24 mm^−^^1^
*c* = 41.397 (3) Å	*T* = 296 K
*V* = 3516.2 (4) Å^3^	Needle, colorless
*Z* = 8	0.45 × 0.20 × 0.05 mm
*F*(000) = 1536	

### Data collection

**Table:**

STOE IPDS 2 diffractometer	2913 independent reflections
Radiation source: sealed X-ray tube, 12 x 0.4 mm long-fine focus	1682 reflections with *I* > 2σ(*I*)
Plane graphite monochromator	*R*_int_ = 0.113
Detector resolution: 6.67 pixels mm^-1^	θ_max_ = 24.5°, θ_min_ = 2.0°
rotation method scans	*h* = −8→8
Absorption correction: integration (X-RED32; Stoe & Cie, 2002)	*k* = −13→13
*T*_min_ = 0.925, *T*_max_ = 0.994	*l* = −48→48
19519 measured reflections	

### Refinement

**Table:**

Refinement on *F*^2^	Primary atom site location: structure-invariant direct methods
Least-squares matrix: full	Secondary atom site location: difference Fourier map
*R*[*F*^2^ > 2σ(*F*^2^)] = 0.060	Hydrogen site location: inferred from neighbouring sites
*wR*(*F*^2^) = 0.128	H-atom parameters constrained
*S* = 0.99	*w* = 1/[σ^2^(*F*_o_^2^) + (0.0518*P*)^2^] where *P* = (*F*_o_^2^ + 2*F*_c_^2^)/3
2913 reflections	(Δ/σ)_max_ < 0.001
235 parameters	Δρ_max_ = 0.34 e Å^−^^3^
0 restraints	Δρ_min_ = −0.22 e Å^−^^3^

### Special details

**Table:**

Geometry. All esds (except the esd in the dihedral angle between two l.s. planes) are estimated using the full covariance matrix. The cell esds are taken into account individually in the estimation of esds in distances, angles and torsion angles; correlations between esds in cell parameters are only used when they are defined by crystal symmetry. An approximate (isotropic) treatment of cell esds is used for estimating esds involving l.s. planes.

### Fractional atomic coordinates and isotropic or equivalent isotropic displacement parameters (Å^2^)

**Table:**

	*x*	*y*	*z*	*U*_iso_*/*U*_eq_	
Cl1	0.40565 (17)	0.91913 (10)	0.94188 (2)	0.0775 (4)	
O2	0.3849 (3)	0.58839 (19)	0.76710 (5)	0.0548 (6)	
O1	0.3725 (4)	0.5145 (2)	0.82642 (5)	0.0652 (7)	
O3	0.4691 (5)	0.8576 (2)	0.48754 (6)	0.0901 (11)	
N1	0.4362 (5)	0.9053 (3)	0.57229 (6)	0.0583 (9)	
N2	0.4339 (5)	0.9186 (3)	0.53853 (6)	0.0627 (9)	
H2	0.420683	0.987853	0.531280	0.075*	
C9	0.3861 (5)	0.6229 (3)	0.73520 (7)	0.0446 (8)	
C12	0.3776 (5)	0.6716 (3)	0.66888 (7)	0.0450 (8)	
C7	0.3875 (5)	0.6191 (3)	0.82384 (7)	0.0459 (8)	
C14	0.4262 (5)	0.7332 (3)	0.72432 (7)	0.0460 (9)	
H14	0.455198	0.791510	0.738937	0.055*	
C15	0.3713 (5)	0.6918 (3)	0.63384 (7)	0.0485 (9)	
H15	0.336983	0.628939	0.621208	0.058*	
C8	0.4077 (5)	0.6746 (3)	0.79090 (7)	0.0463 (8)	
H8A	0.316866	0.734746	0.788149	0.056*	
H8B	0.527120	0.709594	0.788902	0.056*	
C10	0.3420 (5)	0.5366 (3)	0.71335 (8)	0.0493 (9)	
H10	0.315275	0.462357	0.720621	0.059*	
C6	0.3939 (5)	0.6959 (3)	0.85261 (7)	0.0442 (8)	
C13	0.4229 (4)	0.7563 (3)	0.69135 (8)	0.0480 (9)	
H13	0.451610	0.830326	0.684145	0.058*	
C11	0.3378 (5)	0.5613 (3)	0.68064 (8)	0.0495 (9)	
H11	0.307759	0.502891	0.666138	0.059*	
C17	0.4022 (5)	0.8025 (3)	0.58300 (7)	0.0480 (9)	
C16	0.4088 (5)	0.7891 (3)	0.61803 (8)	0.0513 (9)	
H16	0.441662	0.853378	0.630205	0.062*	
C5	0.3951 (5)	0.6460 (3)	0.88335 (7)	0.0505 (9)	
H5	0.395053	0.565786	0.885305	0.061*	
C1	0.3956 (5)	0.8158 (3)	0.85007 (8)	0.0507 (9)	
H1	0.393722	0.850303	0.829780	0.061*	
C4	0.3965 (5)	0.7130 (3)	0.91071 (8)	0.0546 (10)	
H4	0.394802	0.678837	0.931046	0.066*	
C3	0.4005 (5)	0.8324 (3)	0.90763 (8)	0.0542 (9)	
C2	0.4002 (5)	0.8843 (3)	0.87751 (8)	0.0560 (10)	
H2A	0.403021	0.964539	0.875701	0.067*	
C18	0.3570 (6)	0.7050 (3)	0.56077 (8)	0.0638 (11)	
H18A	0.388487	0.632317	0.571073	0.077*	
H18B	0.227071	0.704664	0.556707	0.077*	
C20	0.4501 (6)	0.8347 (4)	0.51664 (9)	0.0667 (12)	
C19	0.4555 (7)	0.7143 (3)	0.52951 (9)	0.0770 (14)	
H19A	0.400668	0.662153	0.513941	0.092*	
H19B	0.581099	0.690928	0.532538	0.092*	

### Atomic displacement parameters (Å^2^)

**Table:**

	*U*^11^	*U*^22^	*U*^33^	*U*^12^	*U*^13^	*U*^23^
Cl1	0.1050 (9)	0.0789 (7)	0.0487 (5)	−0.0053 (7)	−0.0003 (6)	−0.0147 (5)
O2	0.0842 (18)	0.0491 (13)	0.0311 (12)	−0.0041 (14)	−0.0035 (13)	0.0037 (11)
O1	0.102 (2)	0.0493 (16)	0.0440 (14)	−0.0081 (15)	0.0098 (15)	0.0043 (12)
O3	0.168 (3)	0.0733 (19)	0.0293 (15)	−0.019 (2)	0.0040 (16)	0.0027 (13)
N1	0.089 (3)	0.058 (2)	0.0279 (14)	−0.0069 (18)	0.0018 (15)	0.0022 (14)
N2	0.101 (3)	0.0548 (18)	0.0319 (15)	−0.0002 (18)	−0.0013 (16)	0.0067 (15)
C9	0.051 (2)	0.052 (2)	0.0315 (17)	−0.0038 (18)	0.0023 (17)	0.0030 (16)
C12	0.050 (2)	0.051 (2)	0.0340 (17)	−0.0002 (18)	0.0026 (16)	−0.0011 (16)
C7	0.051 (2)	0.050 (2)	0.0368 (18)	−0.0005 (18)	0.0030 (18)	0.0056 (15)
C14	0.054 (2)	0.048 (2)	0.0361 (18)	−0.0049 (18)	−0.0008 (16)	0.0006 (16)
C15	0.057 (2)	0.056 (2)	0.0321 (17)	−0.0005 (19)	0.0012 (18)	−0.0004 (16)
C8	0.056 (2)	0.048 (2)	0.0347 (17)	−0.0009 (19)	0.0018 (17)	0.0015 (16)
C10	0.066 (3)	0.0399 (19)	0.0417 (19)	−0.0040 (17)	0.0017 (17)	0.0058 (16)
C6	0.049 (2)	0.049 (2)	0.0346 (17)	0.0001 (18)	0.0049 (17)	0.0051 (15)
C13	0.054 (2)	0.048 (2)	0.0414 (19)	−0.0020 (19)	0.0011 (17)	0.0063 (16)
C11	0.064 (2)	0.049 (2)	0.0351 (18)	−0.0017 (18)	0.0034 (16)	−0.0031 (17)
C17	0.057 (2)	0.053 (2)	0.0349 (17)	0.0009 (19)	−0.0002 (18)	−0.0021 (16)
C16	0.060 (2)	0.059 (2)	0.0347 (18)	−0.004 (2)	0.0018 (19)	−0.0018 (16)
C5	0.063 (2)	0.047 (2)	0.0409 (19)	−0.0010 (19)	−0.0006 (19)	0.0083 (16)
C1	0.070 (3)	0.047 (2)	0.0348 (18)	0.005 (2)	0.0027 (19)	0.0076 (16)
C4	0.069 (3)	0.058 (2)	0.0367 (19)	−0.003 (2)	−0.0005 (19)	0.0076 (16)
C3	0.060 (2)	0.063 (2)	0.0389 (19)	0.002 (2)	0.0005 (19)	−0.0031 (18)
C2	0.071 (3)	0.047 (2)	0.050 (2)	0.001 (2)	0.002 (2)	0.0007 (18)
C18	0.097 (3)	0.057 (2)	0.037 (2)	−0.008 (2)	0.004 (2)	−0.0009 (18)
C20	0.102 (4)	0.065 (3)	0.034 (2)	−0.011 (2)	0.002 (2)	0.001 (2)
C19	0.126 (4)	0.062 (3)	0.043 (2)	−0.006 (3)	0.011 (2)	−0.001 (2)

### Geometric parameters (Å, º)

**Table:**

C20—O3	1.241 (4)	C10—C11	1.384 (5)
N2—C20	1.333 (5)	C10—H10	0.9300
N1—N2	1.406 (4)	C6—C1	1.389 (5)
N1—C17	1.292 (4)	C6—C5	1.397 (4)
C16—C17	1.459 (4)	C13—H13	0.9300
C15—C16	1.329 (5)	C11—H11	0.9300
C12—C15	1.470 (4)	C17—C18	1.492 (5)
C7—O1	1.219 (4)	C16—H16	0.9300
Cl1—C3	1.737 (3)	C5—C4	1.373 (5)
O2—C9	1.379 (4)	C5—H5	0.9300
O2—C8	1.411 (4)	C1—C2	1.385 (5)
N2—H2	0.8600	C1—H1	0.9300
C9—C14	1.384 (4)	C4—C3	1.386 (5)
C9—C10	1.385 (4)	C4—H4	0.9300
C12—C13	1.391 (4)	C3—C2	1.383 (5)
C12—C11	1.395 (5)	C2—H2A	0.9300
C7—C6	1.486 (4)	C18—C19	1.487 (5)
C7—C8	1.514 (4)	C18—H18A	0.9700
C14—C13	1.391 (4)	C18—H18B	0.9700
C14—H14	0.9300	C20—C19	1.490 (5)
C15—H15	0.9300	C19—H19A	0.9700
C8—H8A	0.9700	C19—H19B	0.9700
C8—H8B	0.9700		
			
C9—O2—C8	117.6 (2)	C12—C11—H11	119.2
C17—N1—N2	116.0 (3)	N1—C17—C16	115.6 (3)
C20—N2—N1	126.5 (3)	N1—C17—C18	121.7 (3)
C20—N2—H2	116.7	C16—C17—C18	122.7 (3)
N1—N2—H2	116.7	C15—C16—C17	124.9 (3)
O2—C9—C14	125.4 (3)	C15—C16—H16	117.5
O2—C9—C10	114.6 (3)	C17—C16—H16	117.5
C14—C9—C10	120.0 (3)	C4—C5—C6	121.2 (3)
C13—C12—C11	117.4 (3)	C4—C5—H5	119.4
C13—C12—C15	123.8 (3)	C6—C5—H5	119.4
C11—C12—C15	118.9 (3)	C2—C1—C6	120.5 (3)
O1—C7—C6	121.7 (3)	C2—C1—H1	119.7
O1—C7—C8	120.5 (3)	C6—C1—H1	119.7
C6—C7—C8	117.8 (3)	C5—C4—C3	119.1 (3)
C9—C14—C13	119.5 (3)	C5—C4—H4	120.5
C9—C14—H14	120.3	C3—C4—H4	120.5
C13—C14—H14	120.3	C2—C3—C4	121.0 (3)
C16—C15—C12	127.9 (3)	C2—C3—Cl1	119.1 (3)
C16—C15—H15	116.1	C4—C3—Cl1	120.0 (3)
C12—C15—H15	116.1	C3—C2—C1	119.4 (3)
O2—C8—C7	108.5 (3)	C3—C2—H2A	120.3
O2—C8—H8A	110.0	C1—C2—H2A	120.3
C7—C8—H8A	110.0	C19—C18—C17	112.0 (3)
O2—C8—H8B	110.0	C19—C18—H18A	109.2
C7—C8—H8B	110.0	C17—C18—H18A	109.2
H8A—C8—H8B	108.4	C19—C18—H18B	109.2
C11—C10—C9	119.7 (3)	C17—C18—H18B	109.2
C11—C10—H10	120.1	H18A—C18—H18B	107.9
C9—C10—H10	120.1	O3—C20—N2	121.0 (4)
C1—C6—C5	118.7 (3)	O3—C20—C19	122.9 (4)
C1—C6—C7	122.4 (3)	N2—C20—C19	116.0 (3)
C5—C6—C7	118.9 (3)	C18—C19—C20	111.4 (3)
C12—C13—C14	121.7 (3)	C18—C19—H19A	109.3
C12—C13—H13	119.1	C20—C19—H19A	109.3
C14—C13—H13	119.1	C18—C19—H19B	109.3
C10—C11—C12	121.7 (3)	C20—C19—H19B	109.3
C10—C11—H11	119.2	H19A—C19—H19B	108.0
			
C17—N1—N2—C20	−19.7 (6)	N2—N1—C17—C16	178.7 (3)
C8—O2—C9—C14	7.0 (5)	N2—N1—C17—C18	−2.0 (5)
C8—O2—C9—C10	−172.8 (3)	C12—C15—C16—C17	179.1 (4)
O2—C9—C14—C13	179.9 (3)	N1—C17—C16—C15	177.3 (4)
C10—C9—C14—C13	−0.4 (5)	C18—C17—C16—C15	−1.9 (6)
C13—C12—C15—C16	1.2 (6)	C1—C6—C5—C4	0.5 (6)
C11—C12—C15—C16	−178.2 (4)	C7—C6—C5—C4	−178.4 (3)
C9—O2—C8—C7	175.7 (3)	C5—C6—C1—C2	0.6 (6)
O1—C7—C8—O2	6.4 (5)	C7—C6—C1—C2	179.5 (3)
C6—C7—C8—O2	−175.9 (3)	C6—C5—C4—C3	−1.3 (6)
O2—C9—C10—C11	179.6 (3)	C5—C4—C3—C2	1.0 (6)
C14—C9—C10—C11	−0.1 (5)	C5—C4—C3—Cl1	−179.1 (3)
O1—C7—C6—C1	−174.5 (4)	C4—C3—C2—C1	0.1 (6)
C8—C7—C6—C1	7.8 (5)	Cl1—C3—C2—C1	−179.8 (3)
O1—C7—C6—C5	4.4 (6)	C6—C1—C2—C3	−0.9 (6)
C8—C7—C6—C5	−173.3 (3)	N1—C17—C18—C19	33.8 (6)
C11—C12—C13—C14	−0.9 (5)	C16—C17—C18—C19	−147.0 (4)
C15—C12—C13—C14	179.7 (3)	N1—N2—C20—O3	−170.9 (4)
C9—C14—C13—C12	0.9 (5)	N1—N2—C20—C19	5.5 (6)
C9—C10—C11—C12	0.2 (6)	C17—C18—C19—C20	−44.6 (5)
C13—C12—C11—C10	0.3 (5)	O3—C20—C19—C18	−156.4 (4)
C15—C12—C11—C10	179.8 (3)	N2—C20—C19—C18	27.3 (6)

### Hydrogen-bond geometry (Å, º)

*Cg*3 is the centroid of the C9–C14 ring.

**Table:**

*D*—H···*A*	*D*—H	H···*A*	*D*···*A*	*D*—H···*A*
N2—H2···O3^i^	0.86	2.11	2.891 (4)	151
C4—H4···O3^ii^	0.93	2.44	3.327 (4)	160
C13—H13···O1^iii^	0.93	2.53	3.421 (4)	161
C18—H18*A*···Cl1^iv^	0.97	2.94	3.737 (3)	140
C8—H8*B*···*Cg*3^v^	0.97	2.73	3.514 (3)	138

Symmetry codes: (i) −*x*+1, −*y*+2, −*z*+1; (ii) *x*, −*y*+3/2, *z*+1/2; (iii) −*x*+1, *y*+1/2, −*z*+3/2; (iv) −*x*+1, *y*−1/2, −*z*+3/2; (v) *x*−1/2, *y*, −*z*+3/2.
